# Renal denervation reduces left ventricular mass in patients with resistant hypertension - results from a multicenter CMR-study

**DOI:** 10.1186/1532-429X-15-S1-E108

**Published:** 2013-01-30

**Authors:** Felix Mahfoud, Daniel Urban, Desiree C Teller, Christian Ukena, Peter Fries, Günther Schneider, Rolf Gebker, Christopher Schneeweis, Philipp Stawowy, Markus P Schlaich, Murray D Esler, Eckart Fleck, Michael Böhm, Sebastian Kelle

**Affiliations:** 1 Internal Medicine/Cardiology, German Heart Institute Berlin, Berlin, Germany; 2 Klinik für Innere Medizin III, Universitätsklinikum des Saarlandes, Homburg/Saar, Germany; 3 Neurovascular Hypertension & Kidney Disease Laboratory, Baker IDI Heart & Diabetes Institute and Heart Centre, Alfred Hospital, Melbourne, VIC, Australia

## Background

Left ventricular (LV) hypertrophy is a common finding in patients with resistant hypertension and is associated with increased sympathetic activity and high cardiovascular risk. Catheter-based renal denervation (RD) has been shown to reduce blood pressure (BP) and sympathetic tone. The present study aimed to investigate the effect of RD on left ventricular mass, assessed by cardiac magnetic resonance (CMR), in patients with resistant hypertension compared to a control group of medical treated patients.

## Methods

CMR was performed in 37 patients at baseline and 6 months after RD in a multicenter setting with 9 subjects serving as controls. Resistant hypertension was defined as office systolic BP >160 mmHg and >150 mmHg for patients with type 2 diabetes. Clinical data and CMR results were analyzed blinded at both times. Data were analyzed using the paired or unpaired t-test. All continuous parameters are given as mean + one standard deviation (SD). For all tests, p<0.05 was considered statistically significant. All tests were two-sided.

## Results

Patients were middle aged (63 ± 11 years vs. 70 ± 8 years in the control group), had poorly controlled BP and heavily medicated. RD significantly reduced systolic BP and LV mass indexed to heigt1.7 by 7.7 % (50.83 ± 11.3 g/m1.7 at baseline vs. 46.91 ± 11.5 g/m1.7 after 6 months, p<0.001, N=27). In contrast, LV mass did not change in the control group (42.7 ± 9.8 g/m1.7 at baseline vs. 43.3 ± 9.4 g/m1.7 after 6 months, p = 0.516). Ejection fraction remained constant in the RD group (55.8 ±10.6% vs. 58.7±9.5% at 6 months; p=0.109) as well as in the control group (58.5 ± 8.6% vs. 58.7 ± 10.0%, p=0.873). No significant changes between baseline and 6 months were evident for LV end-systolic volume and LV end-diastolic volume, neither in the RD group (ESV: 83.0 ± 39.0 ml vs. 77.2 ± 35.2 ml; p=0.189 and EDV: 182.0 ± 51.8 ml vs. 180.9 ± 49.2; p=0.796), nor in the controls (ESV: 69.6 ± 39.0 ml vs. 68.4 ± 33.4 ml, P=0.746 and EDV: 160.2 ± 50.2 vs. 158.6 ± 37.3 ml, p=0.812).

## Conclusions

Catheter-based renal denervation significantly reduced left ventricular mass in patients with resistant hypertension, as diagnosed by CMR. This might have important prognostic implications in patients with resistant hypertension and high cardiovascular risk.

## Funding

Medtronic.

**Figure 1:**
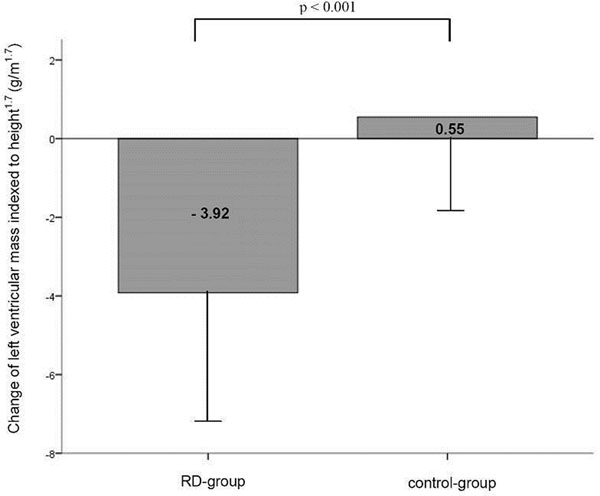
Impact of renal denervation on left ventricular mass (LVM)

